# Liver Fat Is Associated With Elevated FGF21 in Youth With Obesity but Without MASLD

**DOI:** 10.1111/ijpo.70092

**Published:** 2026-02-10

**Authors:** Emir Tas, Eva C. Diaz, Xiawei Ou, Elisabet Børsheim, Silva Arslanian

**Affiliations:** ^1^ UPMC Children's Hospital of Pittsburgh Pittsburgh Pennsylvania USA; ^2^ Center for Pediatric Research in Obesity and Metabolism, Department of Pediatrics University of Pittsburgh Pittsburgh Pennsylvania USA; ^3^ Center for Childhood Obesity Prevention Arkansas Children's Research Institute Little Rock Arkansas USA; ^4^ Arkansas Children's Nutrition Center Little Rock Arkansas USA; ^5^ Department of Pediatrics University of Arkansas for Medical Sciences Little Rock Arkansas USA

**Keywords:** FGF21 (fibroblast growth factor‐21), hepatokine, insulin resistance, liver fat, MASLD, obesity

## Abstract

**Background:**

Youth with obesity are at risk for accumulating liver fat, even below the threshold for metabolic dysfunction‐associated steatotic liver disease (MASLD), defined as ≥ 5% by MRI. While prior studies suggest that sub‐threshold liver fat may carry metabolic risk, the role of fibroblast growth factor‐21 (FGF21)—a liver‐derived hormone responsive to metabolic stress—has not been well characterised in this context.

**Objectives:**

To examine the association between liver fat < 5% and metabolic markers in pubertal youth with obesity, with a focus on FGF21.

**Methods:**

This secondary cross‐sectional analysis included 58 pubertal adolescents with obesity (62% female; mean age 14.7 ± 1.7 years) and liver fat < 5% by MRI‐proton density fat fraction (PDFF). Fasting glucose, insulin, lipids, leptin, adiponectin and FGF21 were measured. Insulin sensitivity was estimated by homeostatic model assessment of insulin resistance (HOMA‐IR). Associations between PDFF and metabolic markers were analysed using Spearman correlations and multivariable regression, adjusted for age, sex, race, ethnicity and body mass index standard deviation score (BMI SDS).

**Results:**

The median PDFF was 2.68%. PDFF correlated positively with BMI SDS, waist circumference, glucose, triglyceride‐to‐high‐density lipoprotein cholesterol ratio (TG/HDL‐C) and FGF21. In adjusted models, PDFF remained independently associated with FGF21 (Beta = 52 pg/mL per 1% increase; *p* = 0.02), even after log transformation. No associations were observed with HOMA‐IR, leptin, or adiponectin.

**Conclusions:**

Among pubertal youth with obesity and liver fat below the MASLD threshold, modest increases in PDFF were independently associated with higher FGF21. These findings support the potential utility of FGF21 as a biomarker of early hepatic‐metabolic stress in the framework of ‘pre‐MASLD’ state—similar to pre‐diabetes before the development of overt steatosis.

## 1 Introduction

The global rise in paediatric obesity is paralleled by an increased prevalence of metabolic comorbidities, including metabolic dysfunction‐associated steatotic liver disease (MASLD) [1]. MASLD affects up to one‐third of children with obesity and is linked to insulin resistance, dyslipidaemia and elevated cardiovascular risk [2, 3]. While a liver fat content of ≥ 5% by magnetic resonance imaging‐proton density fat fraction (MRI‐PDFF) is commonly used to define MASLD, the metabolic implications of liver fat accumulation below this threshold remain poorly understood [2, 4, 5]. Most prior research has focused on youth with established steatosis, limiting insight into the earliest, potentially reversible, stages of hepatic lipid accumulation.

Akin to prediabetes, which represents an early and reversible stage in the development of type 2 diabetes, subclinical elevations in liver fat below the diagnostic threshold for MASLD may signal early hepatic‐metabolic dysfunction. Hepatic fat accumulation is strongly associated with insulin resistance, even when liver fat does not meet diagnostic criteria for MASLD, and may represent one of the earliest signs of metabolic impairment in youth with obesity [6, 7]. Although the clinical significance of this sub‐threshold range remains poorly defined, it may reflect a ‘pre‐MASLD’ state in which hepatic stress precedes overt disease. Identifying such early changes is particularly important in children with obesity, who face an elevated lifetime risk of cardiometabolic complications. Drawing from the prediabetes framework [8], recognising and characterising early hepatic alterations may support more timely risk stratification and prevention. However, few studies have directly examined this subclinical range in paediatric populations.

Fibroblast growth factor 21 (FGF21) is a hepatokine involved in energy balance and nutrient metabolism. It is produced primarily by the liver in response to various metabolic stressors, including hepatic lipid overload, prolonged fasting and mitochondrial dysfunction [9, 10]. FGF21 enhances glucose uptake, stimulates fatty acid oxidation and promotes thermogenesis—serving as an adaptive hormonal response to maintain metabolic homeostasis. Circulating FGF21 is elevated in both paediatric and adult MASLD and is thought to reflect hepatic stress and insulin resistance [11, 12, 13, 14]. In youth, several studies have shown that FGF21 levels correlate with hepatic fat content and metabolic dysfunction, particularly in those with established NAFLD [15, 16, 17]. However, its behaviour at sub‐threshold levels of liver fat remains poorly defined. Some evidence suggests FGF21 may respond to modest hepatic lipid accumulation or increased lipogenesis [16], while other data indicate limited specificity in children without steatosis [17]. This uncertainty limits its current utility as a marker of early hepatic stress. Other metabolic markers—including indices of insulin resistance, circulating adipokines and lipid ratios—though well‐studied in MASLD, may be less sensitive to early hepatic changes.

The aim of this study was to evaluate the relationship between liver fat content and key metabolic markers in youth with obesity but without MASLD. We hypothesised that increasing liver fat, even below the diagnostic threshold, would be associated with early alterations in metabolic markers and that FGF21 may serve as a sensitive biomarker of early hepatic dysfunction. This is, to our knowledge, the first paediatric study to examine the metabolic significance of sub‐threshold liver fat using MRI‐PDFF while specifically excluding MASLD to isolate early hepatic signalling—particularly the role of FGF21—as a potential marker of preclinical metabolic stress.

## 2 Methods

### 2.1 Study Population

This secondary analysis utilised baseline data from two previously published studies conducted at the Arkansas Children's Nutrition Center, both of which used identical imaging protocols and laboratory methods for metabolic phenotyping [18, 19]. The studies were approved by the Institutional Review Board at the University of Arkansas for Medical Sciences (IRB #206278 and #260671), and all participants provided written informed assent with parental consent. One of the original studies was registered at ClinicalTrials.gov (NCT04342390).

The combined cohort included 99 adolescents aged 10–18 years with obesity (body mass index [BMI] ≥ 95th percentile for age and sex) who underwent fasting bloodwork and liver fat quantification using MRI‐PDFF. Self‐reported race and ethnicity were collected via parent or participant report and categorised as Black or White for race, and Hispanic or non‐Hispanic for ethnicity. To isolate the metabolic effects of hepatic fat accumulation below the diagnostic threshold for MASLD, participants with PDFF ≥ 5% were excluded, yielding a final analytic sample of 58 youth with PDFF < 5%. While modest, this sample size is within the range of other paediatric MRI‐PDFF studies and enabled us to isolate a narrowly defined, high‐risk subgroup: pubertal youth with obesity but without MASLD. This focused design, combined with standardised imaging and biomarker protocols, allowed us to detect statistically significant associations with FGF21 despite the limited cohort size.

### 2.2 Anthropometric and Clinical Measures

Height and weight were measured using a calibrated stadiometer and scale, and BMI was calculated as weight in kilograms divided by height in meters squared (kg/m^2^). Body mass index standard deviation scores (BMI SDS) were determined using the Centers for Disease Control and Prevention growth charts [20]. Waist circumference was measured at the level of the iliac crest using a non‐stretchable tape. Blood pressure was measured in the seated position after 15 min of rest using an automated sphygmomanometer.

### 2.3 Laboratory Measures

Fasting blood samples were collected after an overnight fast of at least 10 h. Glucose, total cholesterol, HDL cholesterol, LDL cholesterol, triglycerides and liver enzymes (alanine aminotransferase [ALT] and aspartate aminotransferase [AST]) were measured using either the Siemens Atellica (Malvern, PA) at Arkansas Children's Hospital or the RX Daytona analyzer (Randox Laboratories, Kearneysville, WV) at Arkansas Children's Research Institute.

Serum insulin, leptin, adiponectin and FGF21 were measured using enzyme‐linked immunosorbent assays (ELISA; R&D Systems, Minneapolis, MN) at the Metabolism and Bioenergetics Core at the Arkansas Children's Nutrition Center/Arkansas Children's Research Institute. All samples were analysed in duplicate within a single batch during each study period to minimise variability.

Insulin sensitivity was estimated using the homeostasis model assessment of insulin resistance (HOMA‐IR) formula:

$$\text{HOMA}-\mathrm{IR}=\left[\text{fasting insulin}\;\left(\mathrm{\mu U}/\mathrm{mL}\right)\times \text{fasting glucose}\;\left(\mathrm{mg}/\mathrm{dL}\right)\right]/405$$

### 2.4 Liver Fat Quantification

Liver fat was quantified using MRI‐PDFF on a 1.5 T scanner (Philips Healthcare, Best, Netherlands) as previously described [18, 19]. A multiecho, multislice gradient‐echo pulse sequence was used (TR = 150 ms, flip angle = 25°, echo times = 2.3, 4.6 and 9.2 ms) with breath‐hold acquisition to obtain in‐phase and out‐of‐phase images. A triple‐echo method was employed to minimise confounding from T2 and T1 relaxation effects. Raw MRI data were analysed using customised MATLAB scripts (MathWorks, Natick, MA). Two raters, blinded to participant data, manually drew regions of interest encompassing as much of the liver parenchyma as possible while avoiding large vessels, perihepatic fat and liver edges. PDFF values were calculated based on signal intensity changes across the echo times within each region of interest.

### 2.5 Statistical Analysis

Descriptive statistics were used to characterise the cohort. Continuous variables were assessed for normality using histograms and the Shapiro‐Wilk test. Normally distributed variables are reported as mean ± standard deviation, and non‐normally distributed variables as median (interquartile range). Bivariate associations between liver fat content (PDFF) and metabolic markers were evaluated using Spearman rank correlation coefficients, given the non‐normal distribution of PDFF. PDFF was treated as a continuous independent variable in all regression models.

Multivariable linear regression was used to examine associations between PDFF and metabolic outcomes, including HOMA‐IR, leptin, adiponectin, leptin‐to‐adiponectin ratio, the triglyceride‐to‐HDL cholesterol ratio (TG/HDL‐C) and FGF21. All models were adjusted for age, sex, race, ethnicity and BMI SDS. Multicollinearity was assessed using variance inflation factors (VIF), with all covariates within acceptable limits (VIF < 2). For FGF21, additional sensitivity analyses were performed using log‐transformed values to account for non‐normal distribution. To assess the robustness of the primary findings, a separate sensitivity analysis was performed on a subgroup of participants with PDFF < 4%. Furthermore, post hoc sensitivity analyses in the entire cohort (*n* = 58) were conducted to estimate the minimal detectable partial correlation given the available sample size and covariates (*α* = 0.05, two‐sided, 80% power). Statistical significance was defined as *p* < 0.05. All analyses were conducted using SPSS version 29 (IBM Corp., Armonk, NY, USA); sensitivity analyses were performed using statistical power calculations for linear regression models, with partial correlations as the measure of effect size.

## 3 Results

### 3.1 Participant Characteristics

A total of 58 pubertal youth aged 10–18 years with obesity (62% female, 41% White, 17% Hispanic) and MRI–PDFF < 5% were included in the analysis. The participants' characteristics are listed in Table 1. All participants were pubertal at the time of assessment by Tanner staging, with 19% in early to mid‐puberty (stages II–III) and 81% in late puberty (stages IV–V).

**TABLE 1:** Descriptive characteristics of pubertal youth with obesity and MRI‐PDFF < 5%.

Variable	Value
Physical characteristics	
Age (years)	14.7 ± 1.7
Sex (%, female)	36 (62%)
Race (%, White)	24 (41%)
Ethnicity (%, Hispanic)	10 (17%)
Weight (kg)	92 ± 22
BMI (kg/m^2^)	34.3 ± 5.7
BMI SDS	2.19 ± 0.37
Waist circumference (cm)	111 ± 15
Systolic BP (mmHg)	118 ± 13
Diastolic BP (mmHg)	69 ± 9
Metabolic characteristics	
Glucose (mmol/L)	5.2 ± 0.4
Insulin (pmol/L)	138.9 (84.0–221.0)
HOMA‐IR	4.62 (2.88–7.79)
Triglycerides (mmol/L)	1.13 ± 0.67
Total cholesterol (mmol/L)	4.29 ± 0.83
LDL‐cholesterol (mmol/L)	2.69 ± 0.72
HDL‐cholesterol (mmol/L)	1.19 ± 0.28
Non‐HDL cholesterol (mmol/L)	2.74 ± 0.76
TG/HDL‐C ratio	0.84 (0.54–1.36)
Leptin (pg/mL)	60.2 (32.6–79.4)
Adiponectin (ng/mL)	6.7 (4.8–9.2)
Leptin/adiponectin (pg/ng) ratio	9.3 (4.5–17.2)
Liver characteristics	
ALT (U/L)	24 (17–34)
AST (U/L)	21 (18–27)
FGF21 (ng/L)	134 (87–213)
PDFF	2.68 (2.06–3.90)

*Note:* Data are presented as mean ± standard deviation for normally distributed variables and median (interquartile range) for non‐normally distributed variables.

Abbreviations: ALT, alanine aminotransferase; AST, aspartate aminotransferase; BMI SDS, body mass index standard deviation score; BP, blood pressure; FGF21, fibroblast growth factor 21; HOMA‐IR, homeostatic model assessment of insulin resistance; PDFF, proton density fat fraction; TG/HDL‐C, triglyceride‐to‐high‐density lipoprotein cholesterol.

Liver fat content ranged from 0.75% to 4.9%, with a median PDFF of 2.68% (IQR: 2.06–3.90). The median HOMA‐IR was 4.62 (2.88–7.79), leptin 16.6 μg/L (7.7–32.5), adiponectin 6.7 mg/L (4.8–9.2) and FGF21 134 ng/L (87–213) (Table 1).

Males had a significantly higher PDFF than females (median 4.03% [IQR: 2.39–4.46] vs. 2.60% [IQR: 1.91–3.07]; *p* = 0.007). PDFF did not differ significantly by race (median 2.68% in Black vs. 2.61% in White; *p* = 0.862) or ethnicity (median 2.41% in Hispanic vs. 2.74% in non‐Hispanic participants; *p* = 0.510).

### 3.2 Univariate Correlation Analyses

**FIGURE 1:**
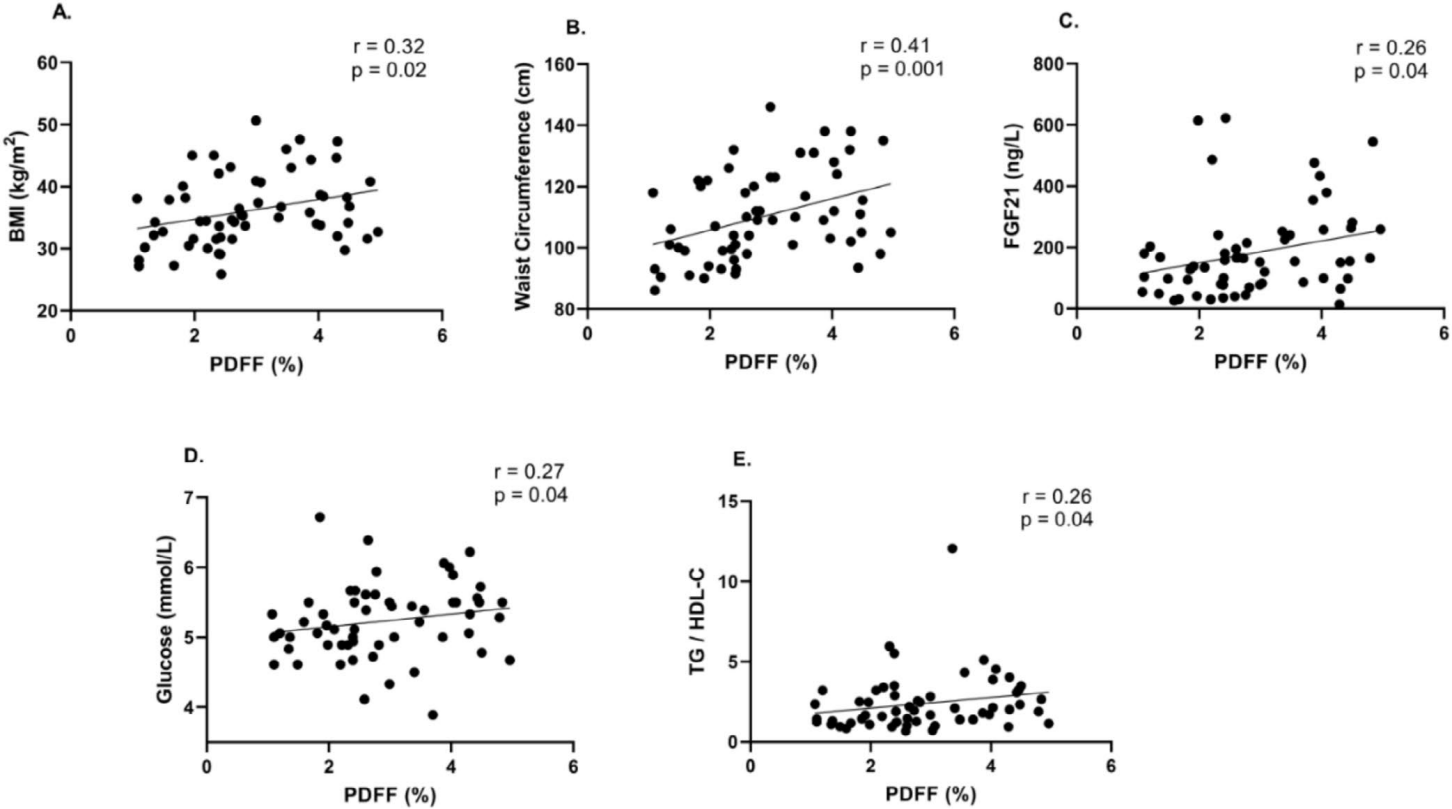
(A) PDFF vs. body mass index (BMI), (B) PDFF vs. waist circumference, (C) PDFF vs. fibroblast growth factor 21 (FGF21), (D) PDFF vs. fasting glucose and (E) PDFF vs. Triglyceride to HDL‐Cholesterol ratio (TG/HDL‐C). Each point represents an individual participant. Spearman correlation coefficients and corresponding *p*‐values are reported on each panel. Regression lines are included for visualisation of linear trends. PDFF, proton density fat fraction.

Spearman rank correlation analyses showed that PDFF was positively correlated with BMI (*ρ* = 0.32, *p* = 0.02) and waist circumference (*ρ* = 0.41, *p* = 0.001), reflecting the relationship between hepatic fat and overall adiposity. PDFF also correlated with FGF21 (*ρ* = 0.26, *p* = 0.04), fasting glucose (*ρ* = 0.27, *p* = 0.04) and TG/HDL‐C ratio (*ρ* = 0.26, *p* = 0.04) (Figure 1). In contrast, PDFF was not significantly correlated with insulin, HOMA‐IR, leptin, adiponectin, leptin/adiponectin ratio or liver enzymes (Table 2).

**TABLE 2:** Spearman correlation between liver fat content (PDFF) and clinical variables.

Variable	Spearman *ρ*	*p*
Age (years)	−0.05	0.69
Systolic BP (mmHg)	0.19	0.15
Diastolic BP (mmHg)	0.08	0.57
Insulin (pmol/L)	0.12	0.36
HOMA‐IR	0.17	0.20
Triglycerides (mmol/L)	0.24	0.07
Total cholesterol (mmol/L)	0.01	0.98
LDL‐cholesterol (mmol/L)	0.05	0.74
HDL‐cholesterol (mmol/L)	−0.18	0.18
Non‐HDL‐cholesterol (mmol/L)	0.10	0.46
ALT (U/L)	0.08	0.58
AST (U/L)	−0.01	0.92
Leptin (pg/mL)	0.09	0.53
Adiponectin (ng/mL)	−0.21	0.12
Leptin/adiponectin ratio (μg/mg)	0.20	0.14

*Note:* Spearman correlation coefficients (ρ) and corresponding *p*‐values for associations between liver fat content (PDFF) and selected clinical, anthropometric and biochemical variables.

Abbreviations: ALT, alanine aminotransferase; AST, aspartate aminotransferase; BMI SDS, body mass index standard deviation score; BP, blood pressure; FGF21, fibroblast growth factor 21; HOMA‐IR, homeostatic model assessment of insulin resistance; PDFF, proton density fat fraction; TG/HDL, triglyceride‐to‐high‐density lipoprotein cholesterol ratio.

### 3.3 Multivariable Linear Regression Models

In models adjusted for age, sex, race, ethnicity and BMI SDS, liver fat content (PDFF) was independently associated with circulating FGF21. Specifically, each 1% increase in PDFF was associated with a 52.1 ng/L increase in FGF21 (95% CI: 7.8 to 96.2; *p* = 0.02). This association remained statistically significant when FGF21 was log‐transformed to account for non‐normal distribution (*B* = 0.13; 95% CI: 0.02 to 0.24; *p* = 0.02). In contrast to FGF21, other markers were not significantly associated with PDFF (Table 3).

**TABLE 3:** Multivariable linear regression: association between PDFF and metabolic markers.

Outcome variable	PDFF coefficient (*B*)	95% CI for *B*	Standardised beta	*p*
FGF21 (ng/L)	52.1	7.8, 96.2	0.38	**0.02**
Log FGF21	0.13	0.02, 0.24	0.39	**0.02**
HOMA‐IR	0.12	−1.22, 1.45	0.03	0.86
Leptin (pg/mL)	4.58	−2.32, 11.47	0.15	0.19
Adiponectin (ng/mL)	−0.78	−1.83, 0.28	−0.24	0.15
TG/HDL‐C ratio	0.29	−0.25, 0.83	0.18	0.29
Leptin/adiponectin ratio	1.32	−0.97, 3.61	0.15	0.25

*Note:* Linear regression models examining the association between liver fat content (PDFF) and metabolic outcomes, adjusted for age, sex, race, ethnicity and BMI SDS. PDFF Coefficients (B) reflect the unit‐based change in each outcome per 1% increase in PDFF. Standardised beta coefficients represent effect sizes in standard deviation units. Significance defined as *p* < 0.05.

### 3.4 Sensitivity Analysis in Youth With PDFF < 4%

To assess whether associations between liver fat and metabolic biomarkers persist at lower levels of hepatic fat accumulation, we conducted a sensitivity analysis restricted to participants with PDFF < 4% (*n* = 45). In this subgroup, PDFF remained significantly associated with both FGF21 (*B* = 100.7 ng/L, 95% CI: 43.4 to 158.1; *p* = 0.001) and log‐transformed FGF21 (*B* = 0.26, 95% CI: 0.12 to 0.39; *p* < 0.001) after adjustment for age, sex and BMI SDS. Due to the limited sample size, race and ethnicity were not included as covariates in this subanalysis. In contrast, PDFF was not significantly associated with leptin, adiponectin, HOMA‐IR, or the TG/HDL‐C ratio (all *p* > 0.15), consistent with findings from the full cohort (Table S1).

### 3.5 Power Analysis

In the full sample (*n* = 58, 5 covariates), post hoc sensitivity analysis indicated that the minimal detectable partial correlation at *α* = 0.05 and 80% power was |*r*| ≈0.37. The observed PDFF–FGF21 association corresponded to a partial *r* ≈0.32 (partial *r* ^2^ ≈0.10; *f* ^2^ ≈0.11), yielding an estimated post hoc power of ~66%. For secondary outcomes (e.g., HOMA‐IR, leptin/adiponectin ratio, TG/HDL‐C), observed partial correlations were below the minimal detectable threshold, and the 95% confidence intervals for these null findings did not exclude moderate associations (upper bounds up to *r* ≈0.40), indicating limited sensitivity to detect smaller effects. See Table S2 for achieved post hoc power for each adjusted association.

## 4 Discussion

In this study of pubertal youth with obesity and MRI‐defined hepatic fat content below the MASLD diagnostic threshold (PDFF < 5%), we found that modest increases in liver fat were independently associated with elevated circulating FGF21 (ng/L), even after adjustment for BMI SDS, age, sex, race and ethnicity. This association persisted following log transformation of FGF21 and was not mirrored by other cardiometabolic markers, including HOMA‐IR, leptin, adiponectin, TG/HDL‐C ratio or leptin‐to‐adiponectin ratio. Our results suggest that FGF21 may serve as a sensitive, liver‐derived biomarker of early metabolic stress—detectable prior to the development of overt hepatic steatosis.

FGF21 is a hormone primarily secreted by the liver in response to metabolic stress, including lipid overload, mitochondrial dysfunction and endoplasmic reticulum stress [21]. It promotes adaptive changes in glucose uptake, fatty acid oxidation and energy expenditure, and has been shown to increase in MASLD, obesity and type 2 diabetes [13, 22, 23]. However, FGF21 resistance—a state in which circulating levels are elevated but tissue responsiveness is impaired—is frequently observed in these conditions [24, 25]. Thus, increasing FGF21 levels may reflect underlying hepatic dysfunction prior to the emergence of systemic metabolic abnormalities.

Our findings are supported by prior paediatric studies demonstrating associations between FGF21 and hepatic‐metabolic dysfunction in youth. Giannini et al. [15] reported elevated FGF21 levels in adolescents with obesity, which correlated with MRI‐estimated hepatic fat content, even at values below the diagnostic threshold. Reinehr et al. [17] found that while FGF21 levels were higher in children with obesity compared to lean peers, they did not differ significantly between those with and without NAFLD by ultrasound—suggesting that FGF21 may reflect broader metabolic stress rather than liver fat alone. Korwutthikulrangsri et al. [26] also reported higher FGF21 concentrations in Thai children with obesity and with insulin resistance and impaired glucose tolerance. Maffeis et al. [16] demonstrated that genetically driven hepatic lipogenesis is associated with higher FGF21, reinforcing its responsiveness to hepatic lipid metabolism even in the absence of steatosis. Our previous work further showed that the FGF21–adiponectin ratio correlates with liver fat accumulation in youth, particularly in those with NAFLD. Collectively, these studies highlight FGF21 as a sensitive but not fully specific marker of hepatic‐metabolic stress. Building on this literature, our study uniquely focused on pubertal youth with obesity and MRI‐PDFF < 5%, excluded MASLD by design and demonstrated that FGF21 may serve as a non‐invasive biomarker of early hepatic dysfunction in this subclinical range.

Prior paediatric studies have shown that liver fat accumulation below the MASLD threshold may still carry metabolic significance. Cohen et al. [6] reported that even low levels of hepatic fat were independently associated with insulin resistance in preschool‐aged children. Similarly, Geurtsen et al. [7] found that school‐aged children with liver fat between 1% and 5% had higher cardiometabolic risk markers, including insulin resistance and dyslipidaemia. While these studies underscore the clinical relevance of sub‐threshold liver fat, neither focused specifically on youth with obesity nor evaluated liver‐derived hormonal signals. By studying a high‐risk, pubertal cohort with obesity and excluding MASLD by MRI‐PDFF, our work extends this literature and introduces FGF21 as a potential biomarker of early hepatic stress. Together, these findings support the emerging concept of a ‘pre‐MASLD’ state—analogous to prediabetes—in which hepatic‐metabolic dysfunction may arise prior to overt steatosis or systemic abnormalities. Although HOMA‐IR, leptin, adiponectin and the TG/HDL ratio have been widely studied in MASLD [27, 28, 29, 30, 31], these markers, after adjusting for covariates, were not significantly associated with PDFF in our cohort. HOMA‐IR reflects systemic insulin resistance and is often elevated in MASLD but may lack sensitivity for detecting early hepatic lipid stress. Leptin and adiponectin—adipokines involved in energy balance and inflammation—have shown mixed associations with liver fat and often correlate more strongly with general adiposity than liver‐specific dysfunction [32, 33, 34]. Similarly, TG/HDL‐C, a surrogate marker of insulin resistance in paediatric populations, was associated with PDFF in univariate analysis but not in adjusted models—suggesting limited sensitivity to early hepatic‐metabolic stress compared to FGF21 [35, 36]. The leptin‐to‐adiponectin ratio, which integrates opposing adipokine signals and has been proposed as a marker of insulin resistance, was also not significantly associated with liver fat content in our cohort [37]. Collectively, these findings suggest that while these markers are informative in more advanced disease, they may be less useful in detecting early hepatic‐metabolic alterations—highlighting FGF21's potential as a more specific biomarker even in the absence of overt hepatic steatosis.

This study has several strengths, including the use of MRI‐PDFF for quantitative liver fat assessment, a well‐characterised pubertal cohort and a broad panel of fasting metabolic biomarkers. By excluding participants with MASLD, we were able to isolate the effects of sub‐threshold hepatic fat variation. The inclusion of only pubertal youth further reduces confounding by developmental stage, although it is worth noting that 19% of participants were in early stages of pubertal development.

Several limitations warrant consideration. The cross‐sectional design limits causal inference. The modest sample size may also limit generalisability and reduce sensitivity to detect weaker associations, particularly for secondary outcomes. Post hoc sensitivity analysis indicated that our study had a minimal detectable partial correlation of |*r*| ≥ 0.37 with 80% power. The observed PDFF–FGF21 association (partial *r* ≈0.32) was statistically significant but corresponded to an achieved power of ~66%. For secondary outcomes, observed partial correlations were below the minimal detectable threshold, and confidence intervals did not exclude moderate effects, indicating that null findings should be interpreted with caution. While MRI‐PDFF offers sensitive, non‐invasive quantification of hepatic fat, it does not provide information on inflammation or fibrosis, and we did not measure visceral adiposity, which may influence FGF21 levels. Additionally, FGF21 was measured at a single time point, and we did not assess receptor sensitivity or downstream signalling. However, all samples were collected under standardised fasting conditions and analysed in a single batch during the course of individual studies to minimise variability. Whether reductions in liver fat in individuals without overt MASLD—via lifestyle or pharmacologic intervention—lead to decreases in FGF21 remains unknown and warrants further study.

The clinical relevance of FGF21 is further underscored by recent phase 2 trials demonstrating that FGF21 analogues, such as efruxifermin [38] and pegozafermin [39], improve hepatic steatosis, fibrosis and lipid profiles in adults with metabolic‐associated steatohepatitis (MASH). While our study focused on FGF21 as a biomarker in youth, these trials underscore its broader role in hepatic‐metabolic regulation and raise the possibility of both diagnostic and therapeutic applications in paediatric populations.

Emerging evidence suggests that early elevations in FGF21 may represent a compensatory response to hepatic fat accumulation—an attempt by the liver to mitigate early metabolic stress before overt dysfunction occurs. This hypothesis mirrors the well‐established model in prediabetes, where compensatory hyperinsulinemia maintains normal glucose levels in the face of increasing insulin resistance. Just as modest elevations in glucose signal an increased risk for type 2 diabetes, modest elevations in liver fat—reflected by increased PDFF—may mark an early stage of MASLD pathogenesis. In this context, the observed association between PDFF and FGF21 in our study underscores the potential utility of FGF21 as an early biomarker. While we cannot infer causality, these findings raise the possibility of a ‘pre‐MASLD’ state, where subtle shifts in liver fat and FGF21 precede overt liver disease—offering a potential window for monitoring and early intervention in at‐risk youth.

In conclusion, liver fat content below the MASLD diagnostic threshold is associated with elevations in circulating FGF21 among pubertal youth with obesity. These findings suggest that even subclinical hepatic fat accumulation may reflect early metabolic stress. FGF21 may serve as a non‐invasive biomarker of early hepatic stress, even before the onset of steatosis or broader metabolic derangements. Future longitudinal studies should evaluate whether FGF21 levels predict MASLD progression or broader cardiometabolic risk in youth.

## Author Contributions

E.T. conceptualised and designed the study, conducted the statistical analysis and drafted the manuscript. E.C.D., X.O. and E.B. were involved in data acquisition and contributed to data interpretation. S.A. provided overall supervision and critical input on study design and data interpretation. All authors reviewed the manuscript, provided critical feedback and approved the final version.

## Funding

The research reported in this publication was supported by the National Institute of General Medical Sciences of the National Institutes of Health under Award Number P20GM109096 and the US Department of Agriculture‐Agricultural Research Service Project USDA/ARS 6026‐10700‐001‐000D. The content is solely the responsibility of the authors and does not necessarily represent the official views of the National Institutes of Health or USDA‐ARS.

## Conflicts of Interest

The authors declare no conflicts of interest.

## Supporting information


**Table S1:** Multivariable linear regression: association between liver fat.
**Table S2:** Post hoc power and sensitivity analysis for adjusted.

## Data Availability

The original contributions presented in this study are included in the article materials and supplementary data. This was a secondary analysis of previously published studies, and further inquiries can be directed to the corresponding author.
