# Supplementary material for: Liver Fat Is Associated With Elevated FGF21 in Youth With Obesity but Without MASLD
Source: Pediatr Obes. 2026 Feb 10;21(2):e70092. doi: 10.1111/ijpo.70092 (PMC12888524; doi:10.1111/ijpo.70092)
Supplement: Supplementary file 1 — Table S1: Multivariable linear regression: association between liver fat. Table S2: Post hoc power and sensitivity analysis for adjusted. [file IJPO-21-e70092-s001.pdf]

Liver Fat is Associated with Elevated FGF21 in Youth with Obesity but Without MASLD

Emir Tas[1-3,] *, Eva C Diaz[3-5], Xiawei Ou[4,5], Elisabet Børsheim[3-5], Silva Arslanian[1-2]

[1] UPMC Children's Hospital of Pittsburgh, 4401 Penn Avenue, Pittsburgh, PA, 15224, USA
[2] Center for Pediatric Research in Obesity and Metabolism, Department of Pediatrics, University of Pittsburgh, 4401 Penn Avenue, Pittsburgh PA, 15224, USA
[3] Center for Childhood Obesity Prevention, Arkansas Children's Research Institute, 13 Children's Way, Little Rock, AR, 72202, USA
[4] Arkansas Children's Nutrition Center, 15 Children's Way, Little Rock, AR, 72202, USA
[5] Department of Pediatrics, University of Arkansas for Medical Sciences, 4301 West Markham Street, Little Rock, AR, 72205, USA

***Corresponding Author:**

Emir Tas, MD
UPMC Children's Hospital of Pittsburgh
4401 Penn Avenue, Faculty Pavilion #8130
Pittsburgh, PA 15224
Phone: 412-692-8688
Fax: 412-692-5834
E-mail: tase2@upmc.edu

Supplementary Table S1. Multivariable Linear Regression: Association Between Liver Fat Content (PDFF) and Metabolic Markers (PDFF <4%)

Outcome Variable	PDFF Coefficient (B)	95% CI for B	Standardized Beta	p-value
FGF21 (ng/L)	100.7	43.4, 158.1	0.54	**0.001**
Log FGF21	0.26	0.12, 0.39	0.57	**<0.001**
HOMA-IR	–0.22	–2.35, 1.91	–0.04	0.84
Leptin (μg/L)	5.57	–4.54, 15.69	0.13	0.27
TG/HDL-C ratio	0.50	–0.36, 1.37	0.20	0.25
Adiponectin (mg/L)	–1.06	–2.57, 0.44	–0.25	0.16
Leptin/Adiponectin (μg/mg)	2.13	-1.44, 5.69	0.17	0.24

Linear regression models examining the association between liver fat content (PDFF) and metabolic outcomes in the subgroup with PDFF <4%, adjusted for age, sex, and BMI SDS. PDFF coefficients (B) reflect the unit-based change in each outcome per 1% increase in PDFF. Standardized beta coefficients represent effect sizes in standard deviation units. Statistically significant associations ($p < 0.05$) were observed only for FGF21 and log-transformed FGF21.

Supplementary Table S2. Post-hoc power and sensitivity analysis for adjusted associations between PDFF and metabolic outcomes (full sample, n = 58)

Outcome	Partial correlation (adjusted)*	95% CI†	p-value	Post-hoc power‡	Within 80% power detection range? §
FGF21	0.32	0.03 to 0.54	0.02	**0.66**	No
HOMA-IR	0.03	−0.25 to 0.30	0.86	0.06	No
Leptin	0.15	−0.12 to 0.40	0.19	0.19	No
Adiponectin	−0.24	−0.47 to 0.05	0.15	0.41	No
TG/HDL-C	0.18	−0.10 to 0.43	0.29	0.25	No
Leptin/Adiponectin	0.15	−0.12 to 0.40	0.25	0.19	No

* From multivariable linear regression models adjusted for age, sex, race, ethnicity, and BMI SDS.
† 95% CIs derived from the model t-statistic with df=51.
‡ Two-sided α=0.05, computed via noncentral t using the observed partial r (achieved/“observed” power for each effect).
§ Minimal detectable partial correlation from post-hoc sensitivity analysis: $|r| \geq 0.37$ for 80% power at α=0.05 (two-sided).
